# Wine Resveratrol: From the Ground Up

**DOI:** 10.3390/nu8040222

**Published:** 2016-04-14

**Authors:** Luigi Bavaresco, Luigi Lucini, Matteo Busconi, Riccardo Flamini, Mirko De Rosso

**Affiliations:** 1 Department of Sustainable Crop Production, Università Cattolica S. Cuore, Piacenza 29122, Italy; matteo.busconi@unicatt.it; 2 Institute of Agricultural and Environmental Chemistry, Università Cattolica S. Cuore, Piacenza 29122, Italy; luigi.lucini@unicatt.it; 3 CREA-Viticulture, Conegliano 31015, Treviso, Italy; riccardo.flamini@crea.gov.it (R.F.); mirko.derosso@gmail.com (M.D.R.)

**Keywords:** resveratrol, wine, grape, nutraceutical, sirtuins

## Abstract

The ability of the grapevine to activate defense mechanisms against some pathogens has been shown to be linked to the synthesis of resveratrol and other stilbenes by the plant (inducible viniferins). Metabolized viniferins may also be produced or modified by extracellular enzymes released by the pathogen in an attempt to eliminate undesirable toxic compounds. Because of the important properties of resveratrol, there is increasing interest in producing wines with higher contents of this compound and a higher nutritional value. Many biotic and abiotic elicitors can trigger the resveratrol synthesis in the berries, and some examples are reported. Under the same elicitation pressure, viticultural and enological factors can substantially affect the resveratrol concentration in the wine. The production of high resveratrol-containing grapes and wines relies on quality-oriented viticulture (suitable terroirs and sustainable cultural practices) and winemaking technologies that avoid degradation of the compound. In general, the oenological practices commonly used to stabilize wine after fermentation do not affect resveratrol concentration, which shows considerable stability. Finally the paper reports on two sirtuin genes (*SIRT*) expressed in grapevine leaves and berries and the role of resveratrol on the deacetylation activity of the encoded enzymes.

## 1. Resveratrol and its Oligomers in Grape

**Figure 1:**
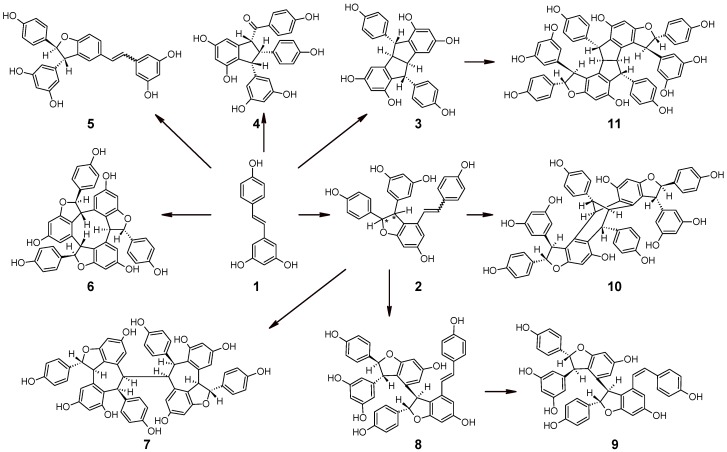
Scheme of formation of viniferins and resveratrol oligomers in grape: (**1**) *trans*-resveratrol; (**2**) (*E* and *Z*) ε-viniferin/ω-viniferin; (**3**) pallidol; (**4**) caraphenol B; (**5**) δ-viniferin (*E* and *Z*); (**6**) α-viniferin; (**7**) isohopeaphenol; (**8**) *E*-miyabenol C; (**9**) *Z*-miyabenol C; (**10**) vaticanol C isomer; and (**11**) ampelopsin H [1].

“Inducible” phytoalexins, such as *trans*-resveratrol and its oligomers (e.g., dimers called viniferins), can be produced in grapevine tissues as an active defense strategy against diseases. On the contrary, “metabolized” stilbenes may be produced by extracellular enzymes released by the pathogen in an attempt to eliminate undesirable toxic compounds. A scheme of formation of resveratrol oligomers in grape is shown in Figure 1 [1].

For example, in *Plasmopara viticola* infected leaves, the enzymes involved in the formation of grape viniferins are expressed both in pathogens and plants [2]. Evidence of the grapevine’s ability to synthesize these compounds is the constitutive presence in different parts of the plant of different stilbenes [3,4] and stilbenoid oligomers [5], which were found in large amounts in vine roots; ε-viniferin was found as a constitutive stilbene of grapevine cluster stems [6], and two δ-viniferin glucosides and pallidol were proved in cell cultures of *Vitis vinifera* Gamay Freaux var. Tenturier [7]. “Inducible” viniferins are hardly detectable in healthy leaves, but their increase in infected leaves was observed.

In *P. viticola* (downy mildew) infected grapevine leaves, the main defense mechanism observed was oxidative dimerisation of resveratrol, even though glycosylation [8], and higher production of ε and δ viniferins was found in resistant varieties. Resveratrol and piceids have low or no toxicity for *P. viticola*, whereas δ-viniferin is highly toxic and can be a resistance marker for this specific disease (downy mildew). In susceptible cultivars, either resveratrol is glycosylated, or its concentration is very low and, consequently, also viniferins are low [8].

In the case of *Botrytis cinerea*, after incubation with resveratrol, the production of six oxidized resveratrol dimers (restrytisols A, B and C, δ-viniferin, leachinol F and pallidol) was observed [9], and the pathogenicity of the different strains (essentially linked to excretion of polyphenoloxidase or laccase enzymes) was correlated to their capacity to degrade the grape viniferins [10,11].

A significant increase in *trans*-resveratrol and oligomers (e.g., caraphenol, *E*-ε-viniferin, ω-viniferin, δ-viniferin, α-viniferin, *E*-miyabenol C, and two tetramers) and a concomitant decrease in glycoside derivatives was observed in Negro Amaro grape berries inoculated by *Aspergillus carbonarius*. Since these resveratrol oligomers were found in both infected and pricked and non-infected berries, they are most likely not “metabolized” compounds but an active defense of the plant against stresses. Their concomitant increase was proposed as a diagnostic marker of the infection [1].

These compounds are synthesized and accumulated into lipid vesicles in the cytoplasm, and then secreted to nearby infected sites in order to limit pathogen proliferation [12], and stilbenes are produced by the phenylpropanoid pathway; stilbene synthase (StSy) is the key enzyme and it produces resveratrol, the basic monomer, which can be glycosylated, hydroxylated, methylated or converted into more complex compounds [13].

The process by which the vine is stimulated to produce secondary metabolites is called “elicitation”, indicating an external stressful stimulus applied to the plant. Besides biotic elicitors, as reported above, also abiotic ones can trigger stilbene synthesis, such as UV (Ultraviolet) irradiation, aluminum chloride, fosetyl-Al, ozone, sucrose, dimethyl-β-cyclodestrin, methyl-jasmonate, benzothiadiazole, chitosan oligomers, salicylic acid, anoxic treatments, abscisic acid (ABA), β-aminobutyric acid (BABA), and emodin [13,14].

Several studies have been conducted on the effects of biotic and abiotic stress conditions on *StSy* gene expression. Recent genome-wide transcriptome analyses on the expression modulation of *StSy* genes at a constitutive level, induced by pathogens and chemical elicitors, have been reported [15].

## 2. Viticultural Factors and Grape Resveratrol

Under the same biotic and/or abiotic elicitation conditions, tissue levels of resveratrol (and its glucoside derivatives—piceids) are affected by the grape variety [16,17], the clone [18], the meteorological conditions [16], the soil type [19] and cultural practices [20,21,22,23].

Resveratrol is present in ripe grapes of both red and white varieties, being higher in the red berries than the white ones [17]. The clone can also play a role, as reported in a pot trial with different clones of Cabernet Sauvignon [18]. Cooler, as opposed to warmer, conditions during ripening, over several years, increase resveratrol grapes concentrations; this is also true for higher vineyard elevation [16]. Calcareous and alkaline, as opposed to non-calcareous and neutral soil, is favorable for increasing the resveratrol concentration in berries at harvest [19].

Increasing the nitrogen supply has a negative effect on resveratrol levels in berries [20], which explains why vines fertilized at high nitrogen rates are more susceptible to diseases.

The effect of removing leaves at veraison in the cluster zone of three varieties was studied in a field trial over four years [21]. Resveratrol concentration in grapes at harvest was affected in a different way depending on the genotype and the meteorological conditions; in cooler years (during ripening time), leaf removal improved resveratrol values over untreated vines, while in warmer years an opposite pattern occurred.

Cluster thinning improved resveratrol concentration as well as its antioxidant capacity in Barbera wine from the Colli Piacentini production area [22].

Both high crop load *versus* low crop load and irrigation *versus* non-irrigation reduced resveratrol concentrations in wines from Sicily [23].

It is difficult to compare data (from literature) on resveratrol concentration in grapes as affected by biotic/abiotic elicitors and viticultural factors because of different extraction methods and units of measurements [13].

## 3. Oenological Factors and Resveratrol in Wine

Resveratrol is contained in considerably higher amounts in red wines than in white wines because it is mainly present in the berry skin, and white wines are usually produced with no or limited maceration with the pomace. Both *trans-* and *cis*-piceid (resveratrol glucosides) are present in grapes and their hydrolysis, occurring during fermentation, releases *cis-* and *trans*-resveratrol. In addition, *trans*–*cis* isomerization can influence the balance between two resveratrol isomers in wine, and their levels can be affected by light. For example, *trans-*resveratrol is stable for months if protected from the light; however, *cis*-resveratrol is stable only near pH neutrality when completely protected from light [24].

Moreover, the choice of yeast can influence the final content of resveratrol in wine due to the different actions of β-glucosidase enzymes, which transform piceids into resveratrol [25].

To some extent, winemaking practices can also potentially affect resveratrol in wine. In general, the low levels of fining agents usually added to stabilize red wines do not significantly reduce the level of *trans*-resveratrol [26], and it is a relatively stable compound that can remain for years in properly stored wines (*i.e.*, avoiding exposure to excess heat, and presence of normal levels of exogenous antioxidants such as sulfur dioxide) [27]. On the other hand, unusual winemaking processes and ageing can induce relevant losses of resveratrol; for instance, Sherry wines showed great losses of resveratrol due to oxidative phenomena and a combination with acetaldehyde and “flor” biofilm growth [28].

The use of specific post-harvest techniques is also able to modulate resveratrol in grapes. For example, irradiation of grapes with ultraviolet-C light could be particularly favorable for the production of raisin wines (e.g., Amarone della Valpolicella) and it has been demonstrated that this biosynthesis in grapes can be induced during the 2–3 months post harvest [29].

The highest concentration of total resveratrol in wine, according to literature data, is 36 mg/L [15].

## 4. Grapevine Sirtuins

Resveratrol has been found to activate sirtuins in budding yeasts [30], worms (*Caenorhabditis elegans*), flies (*Drosophyla melanogaster*) [31] and other metazoans, mimicking the effects of calorie restriction and extending lifespan. In order to explain this behavior of resveratrol, the xenohormesis hypothesis was described [32] as follows: “sirtuin enzymes evolved early in life’s history to increase somatic maintenance and survival during times of adversity. Primordial species synthesized polyphenolic molecules to stimulate sirtuins during times of stress. Plants have retained this ability. Survival pathways in fungi and animals have retained the ability to respond to plant stress signalling molecules because they provide useful prediction about the state of the environment and/or food supply. This ability would allow organisms to prepare for and survive adversity when they might otherwise perish”.

Members of the sirtuin/SIR2 (Silent Information Regulator 2) protein family are NAD^+^ (Nicotinamide adenine dinucleotide)-dependent histone/protein deacetylases and mono ADP (Adenosine diphosphate)-ribosyltransferases. In eukaryotes, sirtuins affect cellular metabolism, being involved in the regulation of transcriptional repression, recombination, cell division cycle, and microtubule organization [33]. In addition, they have been shown to mediate the effect of calorie restriction, which results in life span extension in yeast [34], worms [35] and flies [36].

The interest in looking for grapevine sirtuins arises from the evidence that some long-living plants, such as eucalyptus, spruce, Scots pine and grapevine, produce resveratrol as a stress-induced compound. A hypothesis to be tested is whether or not resveratrol also has other functions in the plant related to longevity through the activation of the sirtuin genes. Therefore, the first investigation was to look for the sirtuin genes in the grapevine genome.

Recently, two putative sirtuin genes have been identified in the *Vitis vinifera* L. genome. Both of these genes appear to be represented by a single copy. One gene (*SIRT4*), on chromosome 7, encodes a SIRT4-like protein and the other one (*SIRT7*), on chromosome 19, encodes a SIRT7-like protein. The two proteins are characterized by conserved domains that categorize them as class II and class IVb sirtuins, respectively [37].

**Figure 2:**
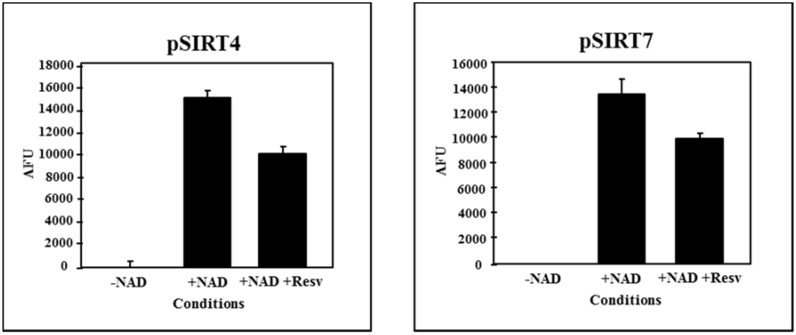
pSIRT4 (SIRT4-like protein) and pSIRT7 (SIRT7-like protein) deacetylation assay in the absence (+NAD) and presence of resveratrol (+NAD + Resv.). Error bars represent SD based on 3 replicates [38].

According to the human SIRT4 and SIRT7 homologues, a very weak NAD^+^-dependent deacetylase activity was detected for both grapevine sirtuin proteins by *in vitro* analysis [38] (Figure 2). No increase in deacetylase activity was detected in the presence of resveratrol (Figure 2), previously reported as a sirtuin activator [30]. Testing ADP ribosyltransferase activity by *in vitro* analysis, lead to no detectable signal on Western blot, suggesting that the NAD^+^ analogue 6-Biotin-17-NAD^+^ did not get covalently incorporated into GDH (Bovine Glutamate Dehydrogenase) the only known human SIRT4 substrate. Therefore, the GDH chosen in the test as the substrate had not been ADP ribosylated by sirtuins. However, this does not completely eliminate the possibility of a ribosyltransferase activity since a NAD^+^ analogue was used. The ribosyltransferase activity of these grapevine proteins, nevertheless, remains an unlikely possibility.

**Figure 3:**
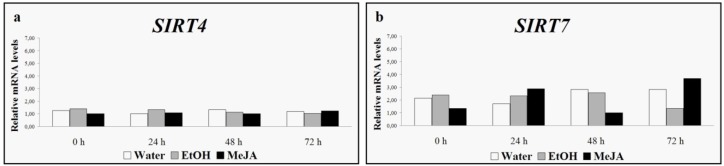
*SIRT4* (**a**) and *SIRT7* (**b**) expression levels induced by water, ethanol (EtOH) and methyl jasmonate (MeJA) [38].

**Figure 4:**
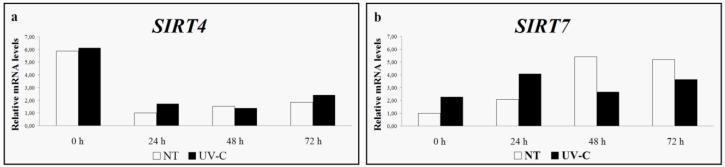
*SIRT4* (**a**) and *SIRT7* (**b**) expression levels in untreated control (NT) and UV-C treated leaves [38].

After a basal transcription observation for both sirtuin genes in cell cultures and leaves, the expression levels of *SIRT4* and *SIRT7* were evaluated under stressful conditions [38]. Our preliminary data showed that neither methyl jasmonate nor UV-C rays influenced the expression of *SIRT4* and *SIRT7* genes (Figure 3 and Figure 4).

Moreover, it was observed, for both sirtuin genes, that a stressful event such as leaf detachment modulated their expression. We can speculate that *SIRT4* expression is quickly down regulated after the stress caused by leaf detachment, while a progressive increment of the *SIRT7* expression seems to be the response to the same stressful event. This unexpected finding is of considerable interest and definitely requires further investigation.

**Figure 5:**
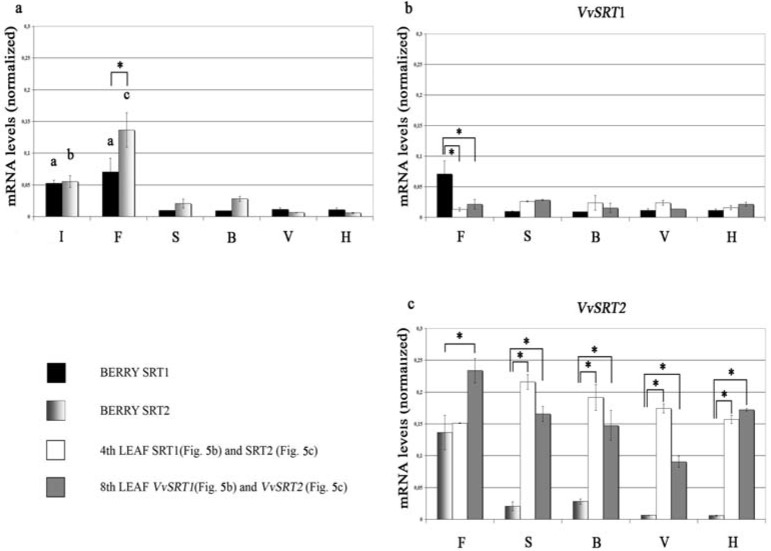
The expression profiles of the two genes in berries are reported. Grapevine growth stages: I, clear inflorescence; F, start of flowering; S, fruit set; B, pea-sized berries; V, veraison; H, harvest. (**a**) reports the comparison between the expression profiles of both genes during the different stages. The two genes have similar expression patterns with the exception of the start of flowering, where the expression level of *VvSRT2* is significantly higher (**c**). The expression level of both genes in the early stages is significantly higher than in the remaining stages: *VvSRT1* (black boxes) shows no difference between clear inflorescence and the start of flowering (**a**), while *VvSRT2* (shaded grey boxes) shows significant differences between clear inflorescence (**b**) and the start of flowering (**c**); (**b**) reports the expression level of *VvSRT1* during the different growing stages in different plant organs: berries (black boxes), fourth leaf (white boxes) and eighth leaf (dark grey boxes); (**c**) Reports the expression level of *VvSRT2* in the different stages and different plant organs: berries (shaded grey boxes), fourth leaf (white boxes) and eighth leaf (dark grey boxes). Error bar indicates ± SD. * *p* < 0.05 (*Post-Hoc* with Bonferroni correction). Reproduced from [39] with permission.

Starting from the predicted coding sequences present in the database, it has been possible to obtain two truly expressed coding sequences from the start to the stop codon for both sirtuin genes that were named *VvSRT1* and *VvSRT2*. In order to better understand the physiological role of both sirtuins, we investigated the expression of these genes in young leaves, mature leaves and berries sampled at different growing stages [39]. In leaves, it was usually observed that *VvSRT1* is less expressed than *VvSRT2*, moreover in young leaves, *VvSRT2* showed the higher expression during fruit set, but during flowering in mature leaves. No particular variations were observed concerning *VvSRT1*. In berries, the two genes showed more similar expression levels, and they showed the highest expression during flowering. Finally, the expression of *VvSRT2* in berries is lower than in leaves (Figure 5).

## 5. Conclusions

The resveratrol concentration in wine is affected by both viticultural and enological factors. Crucial roles are played by the grape variety/clone and the environment; concerning the cultural practices in the vineyard, it can be stated that relying on grape quality standards (no cultivation techniques to force yield) means producing wines with high resveratrol levels. The same goal can be reached in the winery by adopting soft wine making technologies. Finally, resveratrol does not seem to stimulate grapevine sirtuin genes, which are related to the lifespan extension in non-plant organisms.
